# Current coronary intervention controversy: who's to judge?

**DOI:** 10.3402/jchimp.v1i3.7201

**Published:** 2011-10-17

**Authors:** Roger Leonard

**Affiliations:** Montgomery General Hospital, MedStar Health, Inc., Olney, MD, USA

Dear Editor,

The alleged overutilization of coronary interventional procedures by a cardiologist at St. Joseph Medical Center in Maryland has received significant press coverage (1, 2). The issues of appropriate use and patient safety have led to multiple lawsuits on behalf of patients and the issue of defamation of character has resulted in countersuits (3). The possibility of conflict of interest involving the physician and a coronary device manufacturer has been highlighted (4). The hospital has informed and apologized to patients, has resolved regulatory concerns, and has thoroughly restructured its peer review process in the catheterization laboratory.

Who's to judge the complex issue of utilization? The legislators, regulators, health care associations, and medical societies continue to formulate their opinions. The appropriate judge should be the medical profession based on peer review through unbiased clinical analysis and medical ethics. Unfortunately, we abrogated our responsibility long ago and regulators filled the void. Physicians have a vital role to play, but we are not flying alone and do not have the final say.

Déjà vu. More than a decade ago in Redding, California, inappropriate percutaneous coronary intervention (PCI) and coronary artery bypass graft (CABG) procedures were performed on hundreds of intimidated patients while the interventional cardiologist and cardiothoracic surgeon became the rainmakers for the hospital and the hospital became the darling of the for-profit health system, Tenet (5). Bullying of patients, colleagues, and staff coupled with professional acquiescence contributed to non-existent peer review. Ultimately, 42 FBI agents raided Redding Medical Center in October 2002 to secure patient records. Neither Dr. Moon nor Dr. Realyvasquez faced criminal charges; however, Tenet was fined more than $900,000,000 and was happy to reach a global settlement of all claims.

In June, 2011 the US District Judge for the Western District of Tennessee unsealed a whistleblower complaint alleging ‘blatant overutilization of cardiac medical services, including, but not limited to cardiac sonography, scintigraphic stress testing, angiography, angioplasty, and stenting’ at two hospitals in Jackson, Tennessee (6).

What's happening with stents in Maryland? The Maryland Legislature passed a trio of bills to begin to address this issue. SB 960/HB 600 requires expanded sharing of investigatory data to the Office of Health Care Quality, Health Services Cost Review Commission (HSCRC), and any other state or federal investigatory body, as well as permitting regulatory agencies participation in medical review committees. HB 286 will codify many of the procedures under a Joint Committee credentialing process and enhances the internal peer review processes of hospitals by requiring a new practitioner performance evaluation as a condition of licensure. HB 1182 will require a moratorium on establishing a non-primary PCI program until June 30, 2012. Additionally, it will require the Maryland Health Care Commission (MHCC) to study PCI procedures and recommend statutory changes. The Maryland Hospital Association is sharing best practices for revised, internal peer review processes among its membership and establishing principles related to PCI oversight. The American College of Cardiology and the Society for Cardiac Angiography and Intervention recommend that clinical data becomes the filter through which claims data must pass, that options for external peer review become available, and that a study be made to evaluate potential accreditation of catheterization laboratories.

However, coronary stents are only the tip of the regulatory iceberg ahead. The state will be studying regulations concerning the potential overutilization of pacemakers, cardiodefibrillators, joint prostheses, spine surgery, colonoscopies, C-sections, hysterectomies, and diagnostic studies in medical imaging.

How should physicians respond? We must return to our professional roots. The awakening of the medical profession to its clinical and ethical responsibilities is not optional. The vast majority of physicians meet such high standards as individuals. But as a profession, we ease into the trap of being autonomous practitioners who fail to hold our colleagues accountable because the latter is hard work that requires time, integrity, thoroughness, and difficult conversations.

Physicians will be committed to the principle of beneficence by determining best practices and reducing variability of care in holding their colleagues accountable. For coronary stenting, this means applying the appropriate use criteria (AUC) published by the American College of Cardiology (ACC) (7). The incorporation of AUC into the ACC-NCDR (National Cardiovascular Data Registry) for Cath/PCI has recently led to publication of a critical study (8). The NCDR authors report on 500,154 PCIs performed between July 1, 2009 and September 30, 2010 at 1091 US hospitals. Of 355, 417 PCIs done for acute indications, 98.6% were judged to be appropriate. Of 144, 373 PCIs done for non-acute indications, 88.4% were judged appropriate. Of the 11.6% inappropriate non-acute PCIs, there was wide variation among hospitals ranging from 0% to 55%. The majority of inappropriate PCIs were due to the absence of symptoms, low risk stress testing, minimal anti-ischemic therapy, and absence of proximal left anterior descending (LAD) involvement. Individualized hospital reports from NCDR will spark thoughtful local analysis, peer review, and performance improvement.

Physicians will be committed to the principle of autonomy whereby the patient will be better informed of clinical appropriateness, competence, and cost by the physician and the institution. Examples include the mandatory use of the ACC-NCDR Registry for Cath/PCI and Action Registry required by MHCC and the Maryland Hospital Acquired Condition (MHAC) initiative implemented by the HSCRC with public reporting of performance. However, these regulatory actions are controversial because they bear the downside of potential misinformation when claims data and clinical data are mixed with limited physician engagement. In response, the MHCC has convened the Cardiac Data Advisory Committee with strong physician representation and has formed a Technical Advisory Group on Oversight of PCI Services.

Physicians will be committed to effective peer review based on clinical data with the goal of education and performance improvement, not punishment. Physicians will need to engage as a cohesive medical staff working with their hospitals for the betterment of the patient and the community. Medical leadership understands that there is a major cultural change with loss of autonomy in favor of teamwork where communication barriers related to medical hierarchy are removed.

Finally, physicians will understand their vital role in collaborating with legislators, regulators, and hospital administrators. The Maryland Healthcare Education Institute provided a series of conferences on ‘How Do Bad Things Happen?’ The President and CEO of St. Joseph Medical Center gave a detailed report of their work with patients, families, staff, physicians, and regulators at the state and federal levels (9). From adversity, there is much to learn and to share.

‘Who's to judge’ is answered in the plural, not the singular. We will have far greater transparency because it includes the patient. Placing the patient at the center of our vision is essential. After all, we are the patient, too.

*Roger Leonard*, MD Vice President Medical Affairs Montgomery General Hospital MedStar Health, Inc. Olney, MD, USA Email: rfleonard@montgomerygeneral.com
